# Supplementary material for: A Numerical Investigation of Dimensionless Numbers Characterizing Meltpool Morphology of the Laser Powder Bed Fusion Process
Source: Materials (Basel). 2022 Dec 22;16(1):94. doi: 10.3390/ma16010094 (PMC9821554; doi:10.3390/ma16010094)
Supplement: Supplementary file 1 [file materials-16-00094-s001.zip › materials-2058642-supplementary.pdf]

Supplementary Information for "A numerical investigation of dimensionless numbers characterizing meltpool morphology of the laser powder bed fusion process"

Kunal Bhagat, Shiva Rudraraju
Department of Mechanical Engineering, University of Wisconsin-Madison, Madison, WI, USA

Material properties and process variables

Material properties of various alloys and the corresponding AM process variables used in this work are listed here. These properties were collected from multiple sources in the literature. The properties of solid and liquid materials are averaged and dependence on the temperature is neglected.

Table S1: Average material properties for different alloys used to calculate input non-dimensional numbers of the thermo-fluidic model [21], [58]

Property	SS316	Ti6Al4V	IN718	AlSi10Mg	AZ91D
$\rho(\frac{kg}{m^3})$	7800	4000	8100	2670	1675
$c(\frac{J}{kgK})$	490	570	435	890	1122
$k(\frac{W}{mk})$	36.5	7.3	11.4	173.0	77 .5
$\mu(\frac{Kg}{ms})$	7.0×10^{-3}	4.0×10^{-3}	5.0×10^{-3}	1.3×10^{-3}	3.0×10^{-3}
$\frac{d\gamma}{dT}(\frac{N}{mK})$	-4.00×10^{-4}	-2.63×10^{-3}	-3.70×10^{-3}	-3.5×10^{-4}	-2.13×10^{-4}
$\beta(\frac{1}{K})$	5.85×10^{-5}	2.50×10^{-5}	4.8×10^{-5}	2.4×10^{-5}	9.54×10^{-5}
$\kappa(m^2)$	5.56×10^{-13}	5.56×10^{-13}	5.56×10^{-13}	5.56×10^{-13}	5.56×10^{-13}
$L(\frac{J}{kg})$	2.72×10^5	2.84×10^5	2.09×10^5	4.23×10^5	3.73×10^5
T_s(K)	1693	1878	1533	831	743
T_l(K)	1733	1928	1609	867	868

Table S2: Chosen process conditions for different alloys used to calculate input non-dimensional numbers of the thermo-fluidic model

Material	(Laser Power, Scan speed) $(P, \boldsymbol{\nu}_p)$
SS316	(70, 0.3), (80, 0.4), (90, 0.5), (100, 0.6)<br>(110, 0.7), (110, 0.8), (110, 0.9), (110, 1.0)<br>(65, 0.5), (75, 0.5), (85, 0.5), (95, 0.5)
Ti6Al4V	(15, 0.2), (25, 0.5), (35, 0.7), (45, 0.9)<br>(40, 0.6), (40, 0.7), (40, 0.8), (40, 1.0)<br>(35, 0.9), (40, 0.9), (45, 0.9), (50, 0.9)
IN718	(20, 0.15), (30, 0.25), (40, 0.45), (50, 0.75),<br>(45, 0.80), (45, 0.90), (45, 1.0), (45, 1.1)<br>(53, 0.95), (55, 0.95), (58, 0.95), (60, 0.95)
AlSi10Mg	(75, 0.35), (85, 0.45), (95, 0.55), (105, 0.65)<br>(100, 0.6), (100, 0.7), (100, 0.8), (100, 0.9)<br>(90, 1.1), (95, 1.1), (100, 1.1), (110, 1.1)
AZ91D	(35, 0.25), (40, 0.30), (45, 0.35), (50, 0.45<br>(40, 0.30), (40, 0.40), (40, 0.50), (40, 0.60)<br>(40, 0.60), (50, 0.60), (60, 0.60), (70, 0.60)

Additional correlations of the dimensionless numbers

The influence of the Péclet number on advection transport in the meltpool for additional materials IN718 and AZ91D is shown in Figure S1, and the corresponding comparison of the five alloys considered in this work is given in Figure S2. The combined plot of aspect ratio with $\boldsymbol{Ma\hat{U}}$ is given in the Figure S3. The meltpool aspect ratio and Marangoni number of IN718 and Ti6AL4V are similar magnitudes. Similarly, the meltpool aspect ratio and Marangoni number of AZ91D and AlSi10Mg are comparable. The influence of Stefan number on the meltpool volumes for IN718 and AZ91D is shown in Figure S4, and the corresponding comparison of the five alloys considered in this work is given in Figure S5. Influence of dimensionless heat absorbed on non-dimensional temperature gradient (G) for IN718 and AZ91D is shown in Figure S6.

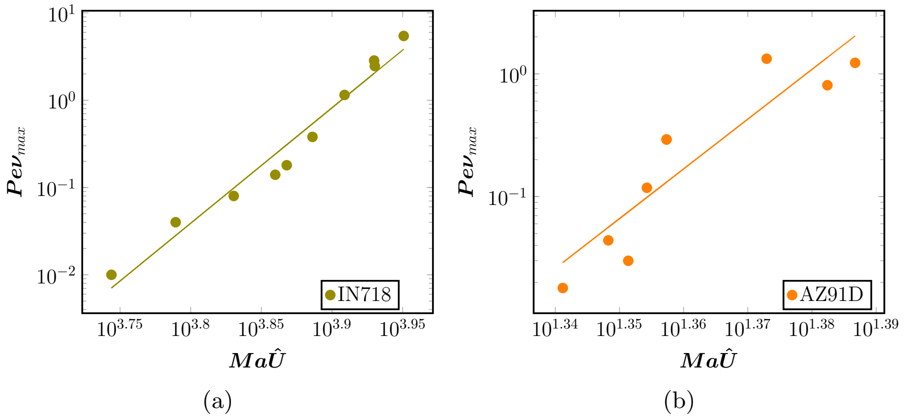

Figure S1: Measure of total advection measured as $\boldsymbol{Pe\nu_{max}}$ vs surface tension based advection $\boldsymbol{Ma\hat{U}} = a_0\boldsymbol{Ma} + a_1\boldsymbol{MaE} + a_2\boldsymbol{MaPe}$ on a log-log scale for (1a)IN718 (1b)AZ91D, alloys.

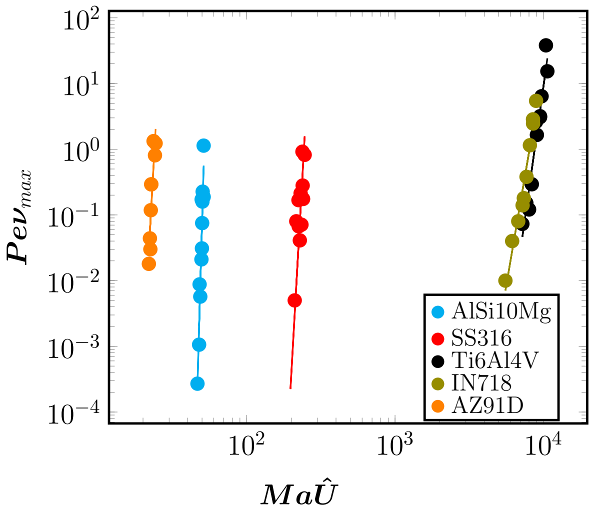

Figure S2: Measure of total advection measured as $\boldsymbol{Pe\nu_{max}}$ vs surface tension based advection $\boldsymbol{Ma\hat{U}} = a_0\boldsymbol{Ma} + a_1\boldsymbol{MaE} + a_2\boldsymbol{MaPe}$, plotted on a log-log scale, for all the five alloys (AlSi10Mg, SS316, Ti6Al4V, IN718 and AZ91D) considered in this work.

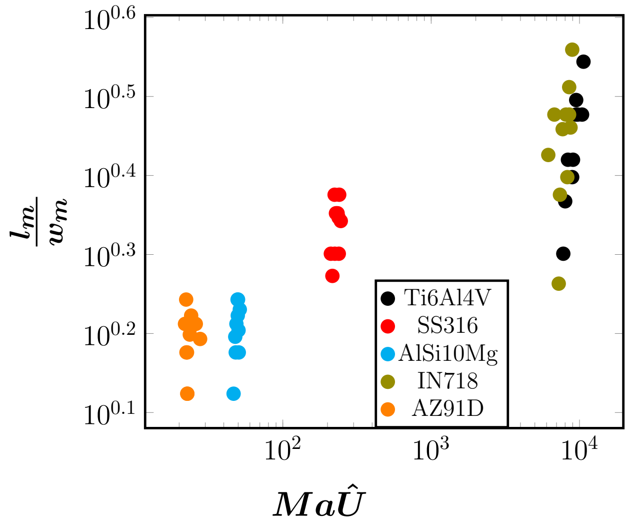

Figure S3: Correlation of the aspect ratio with $\boldsymbol{Ma\hat{U}} = a_0\boldsymbol{Ma} + a_1\boldsymbol{MaE} + a_2\boldsymbol{MaPe}$, plotted on a log-log scale, for all five alloys (Ti6Al4V, SS316, AlSi10Mg, IN718 and AZ91D) shown in a single plot to demonstrate clustering.

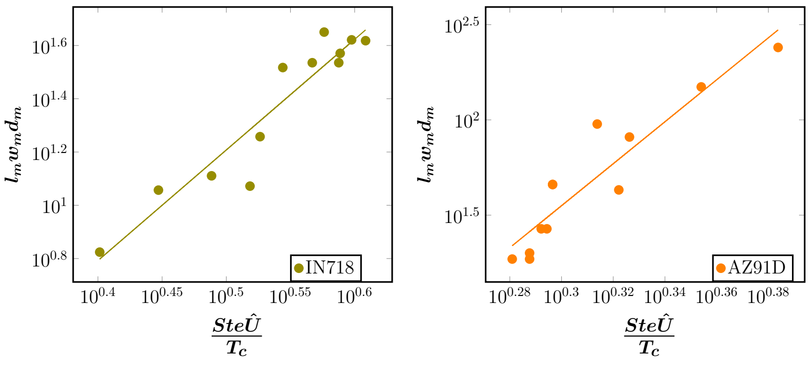

(a) (b)

Figure S4: Correlation of the meltpool volume $(l_m w_m d_m)$ with $\boldsymbol{\frac{Ste\hat{U}}{T_c}} = a_0 \boldsymbol{\frac{Ste}{Tc}} + a_1 \boldsymbol{\frac{SteE}{Tc}} + a_2 \boldsymbol{\frac{StePe}{Tc}}$, plotted on a log-log scale, for (4a) IN718, and (4b) AZ91D, alloys.

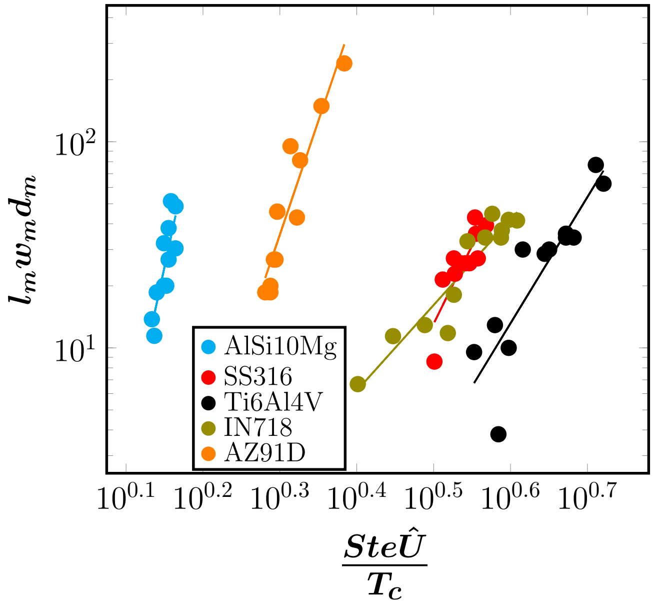

Figure S5: Correlation of the meltpool volume $(l_m w_m d_m)$ with $\boldsymbol{\frac{Ste\hat{U}}{T_c}} = a_0 \boldsymbol{\frac{Ste}{Tc}} + a_1 \boldsymbol{\frac{SteE}{Tc}} + a_2 \boldsymbol{\frac{StePe}{Tc}}$, plotted on a log-log scale, for all the five alloys (AlSi10Mg, SS316, Ti6Al4V, IN718 and AZ91D) considered in this work.

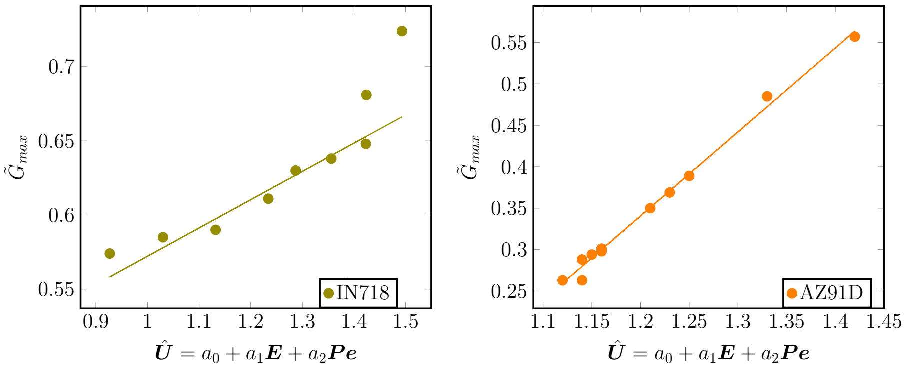

(a) b

(b) b

Figure S6: Dimensionless temperature gradient (G) with $\hat{\boldsymbol{U}}$ for (6a) IN718 and (6b) AZ91D the alloys.
